# Supplementary material for: Sympathetic neuropeptide Y protects from obesity by sustaining thermogenic fat
Source: Nature. 2024 Aug 28;634(8032):243–50. doi: 10.1038/s41586-024-07863-6 (PMC11446830; doi:10.1038/s41586-024-07863-6)
Supplement: Supplementary file 1 — Supplementary Figs. 1–6, Table 1 and additional references. [file 41586_2024_7863_MOESM1_ESM.pdf]

nature portfolio

https://doi.org/10.1038/s41586-024-07863-6

Supplementary information

Sympathetic neuropeptide Y protects from obesity by sustaining thermogenic fat

In the format provided by the authors and unedited

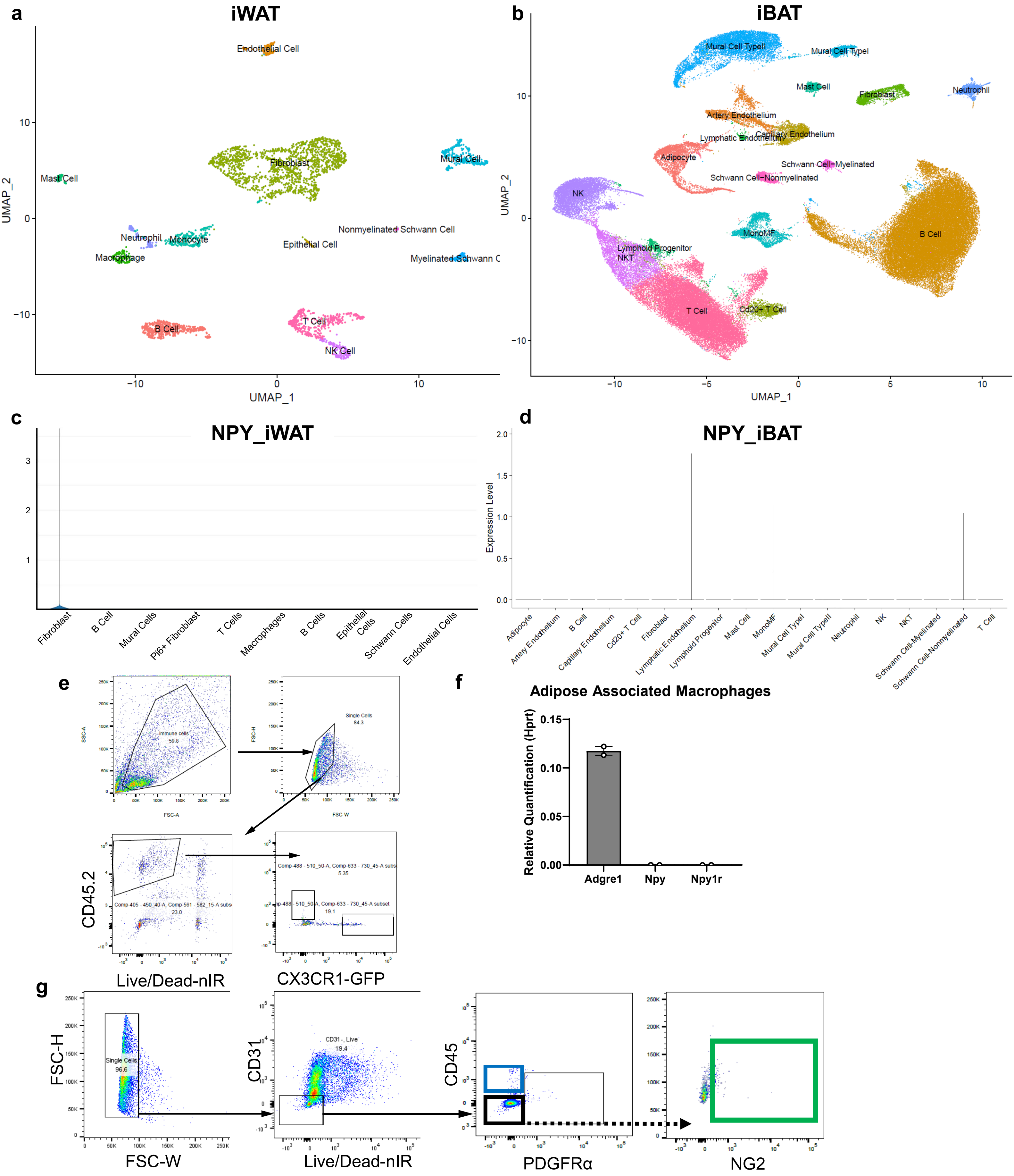

Supplementary Figure 1. *Npy* is not expressed in adipose tissues, and NPY receptors are not expressed in macrophages.

(a-b) Embedding plots showing different clusters of cells in the SVF of murine (a) iWAT[1] and (b) iBAT[2]. **(c-d)** Violin plot showing the expression of *Npy* in the SVF of murine (c) iWAT and (d) iBAT. **(e)** Gating strategy of sorting adipose associated macrophages (ATM) from Cx3cr1$^{GFP/+}$ reporter mice. ATMs were sorted as live/CD45.2^{+}/GFP^{+} cells. **(f)** The expression of *Adgre1* (F4/80), *Npy*, and *Npy1r* in adipose associated macrophages (ATM) (n=2). **(g)** Gating strategy for sorting mural cells from the SVF of the iWAT of wild type normal-diet treated mice, used for Figure 2e. Mural cells (green box) are sorted as live/CD31^{-}/CD45^{-}/PDGFRα^{-}/NG2^{+} cells; immune cells (blue box) are sorted as live, CD31^{-}/CD45^{+} cells. The same gating strategy was also used for cell cytometry data shown in Extended Data Fig. 8c-d. All values are expressed as mean ± SEM.

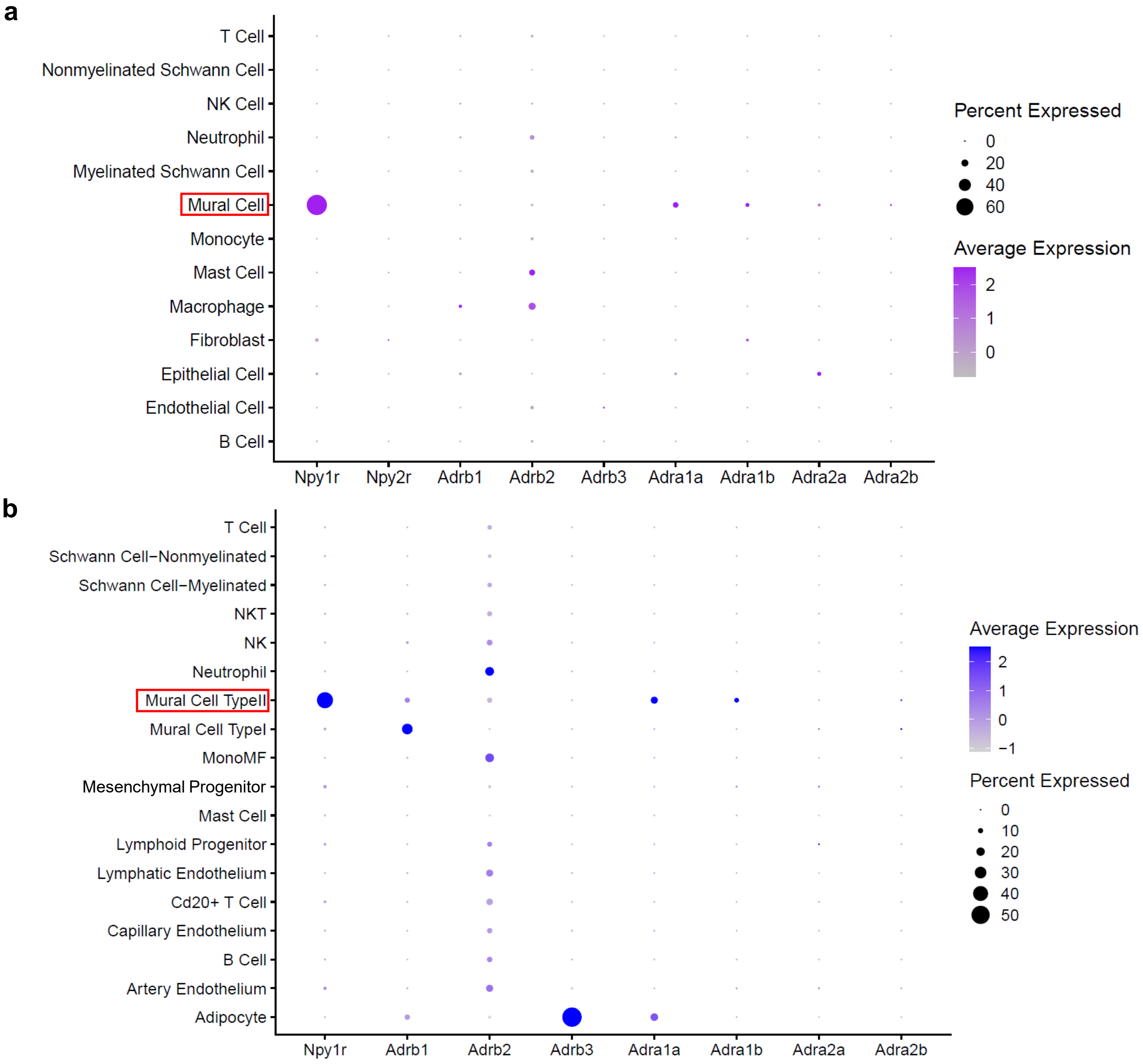

Supplementary Figure 2. The expression levels of *Npy2r* and *Npy5r* in adipose tissues are negligible.
(a) Dot plot showing the expression of *Npy1r*, *Npy2r*, *Adra1a*, *Adra1b*, *Adra2a*, *Adrb1*, *Adrb2*, and *Adrb3* in different cells in the SVF of mice iWAT. **(b)** Dot plot showing the expression of *Npy1r, Adrb1, Adrb2, Adrb3, Adra1a, Adra1b, Adra2a,* and *Adra2b* in different types of cells in mouse iBAT. The size of the dot represents the percentage of cells expressing a certain gene, and the darkness of the dot represents the expression level. Mural cell clusters are highlighted with red rectangles.

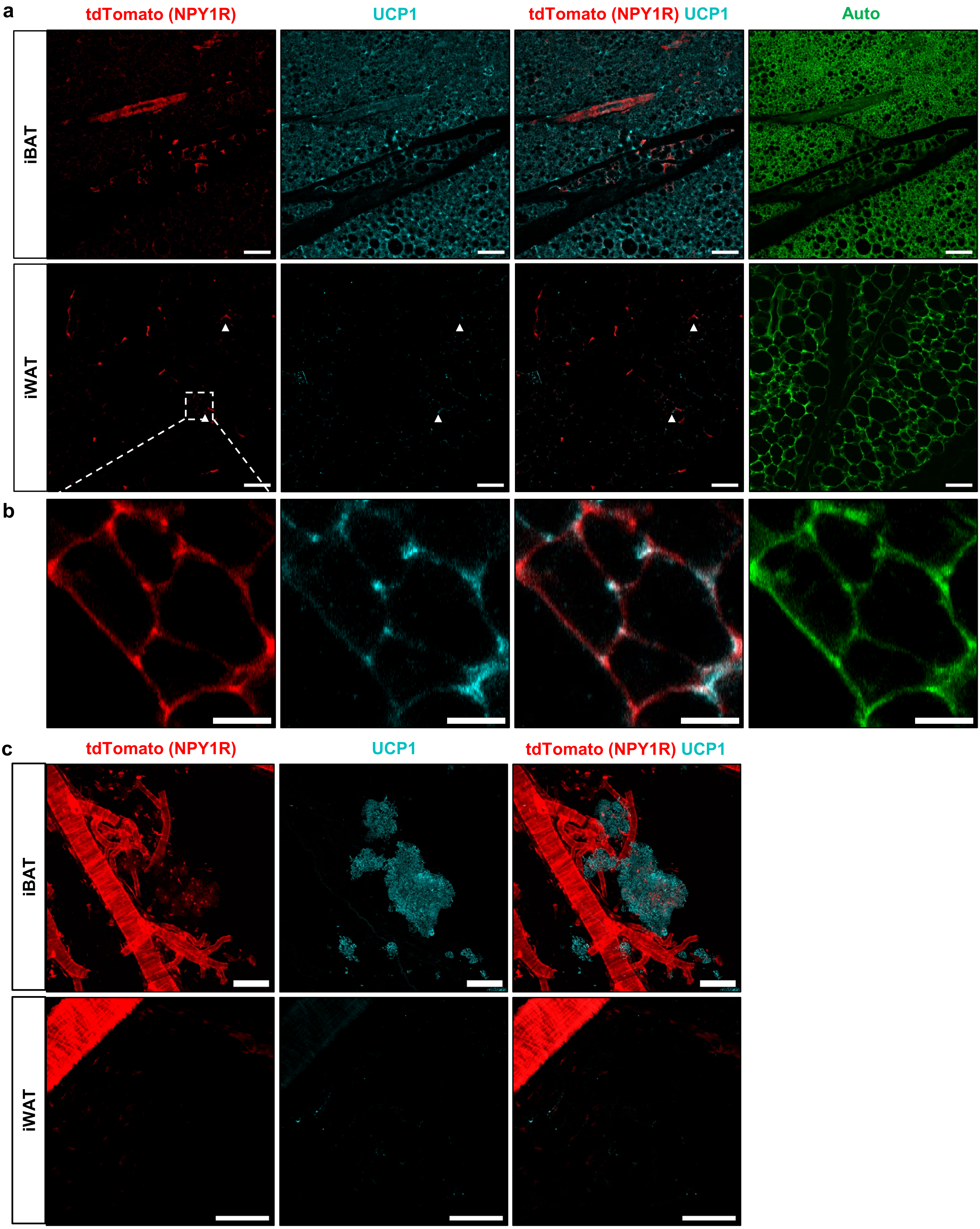

Supplementary Figure 3. Only UCP1⁺ multi-locular adipocytes are lineage-traced to NPY1R.
(a-c) Confocal images of (a) paraffin-embedded slides (scale bar=50μm) and (c) whole-mount vessels (scale bar=100μm) of iBAT and iWAT of 12-week-old male $Npy1r^{Cre}$; $Rosa26^{tdTomato}$ mice stained with UCP1 (Cyan). (b) is zoomed-in (a) (scale bar=10μm). 488nm laser-excited autofluorescence (green), which is deviated from red and far-red (cyan) channels, indicates the presence of adipocytes. White arrows indicate multi-locular $UCP1^{+}$ adipocytes in iWAT. Representative images are shown from 2 experiments.

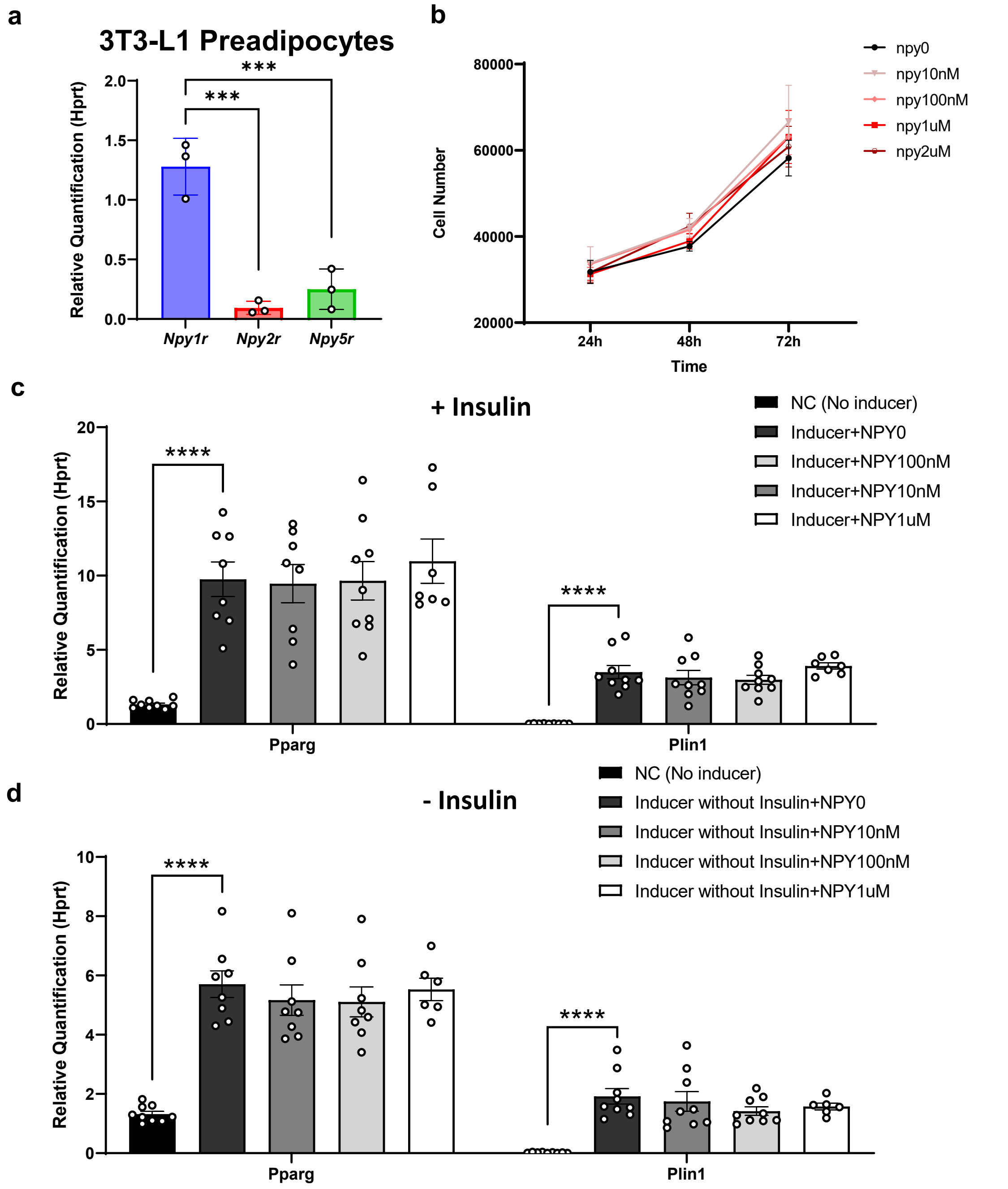

Supplementary Supplementary Figure 4. NPY does not affect proliferation or adipogenesis of 3T3-L1. **(a)** The expression of *Npy1r*, *Npy2r*, and *Npy5r* in 3T3-L1 preadipocyte cell line (n=3 biologically independent samples). *Npy1r* vs. *Npy2r*, P=0.0004; *Npy1r* vs. *Npy5r*, P=0.0008. **(b)** The impact of NPY on the proliferation of 3T3-L1 preadipocytes revealed by the cell number after incubating with or without 1μM NPY for 24, 48, and 72 hours (n=3 biologically independent samples). **(c-d)** The expression of adipogenesis markers *Pparg* and *Plin1* in differentiated 3T3-L1 preadipocytes induced by induction medium (c) with or (d) without 1 μg/mL insulin (n=9, 8, 8, 9 & 7 biologically independent samples per for *Pparg*, n=9, 9, 9, 9 & 7 biologically independent samples per for *Plin1*). The concentration of NPY is indicated in the plot. For all comparisons labelled, P<0.0001. All values are expressed as mean ± SEM. Statistical comparisons were made using one-way ANOVA, *p<0.05, **p<0.01, ***p<0.001, ****p<0.0001.

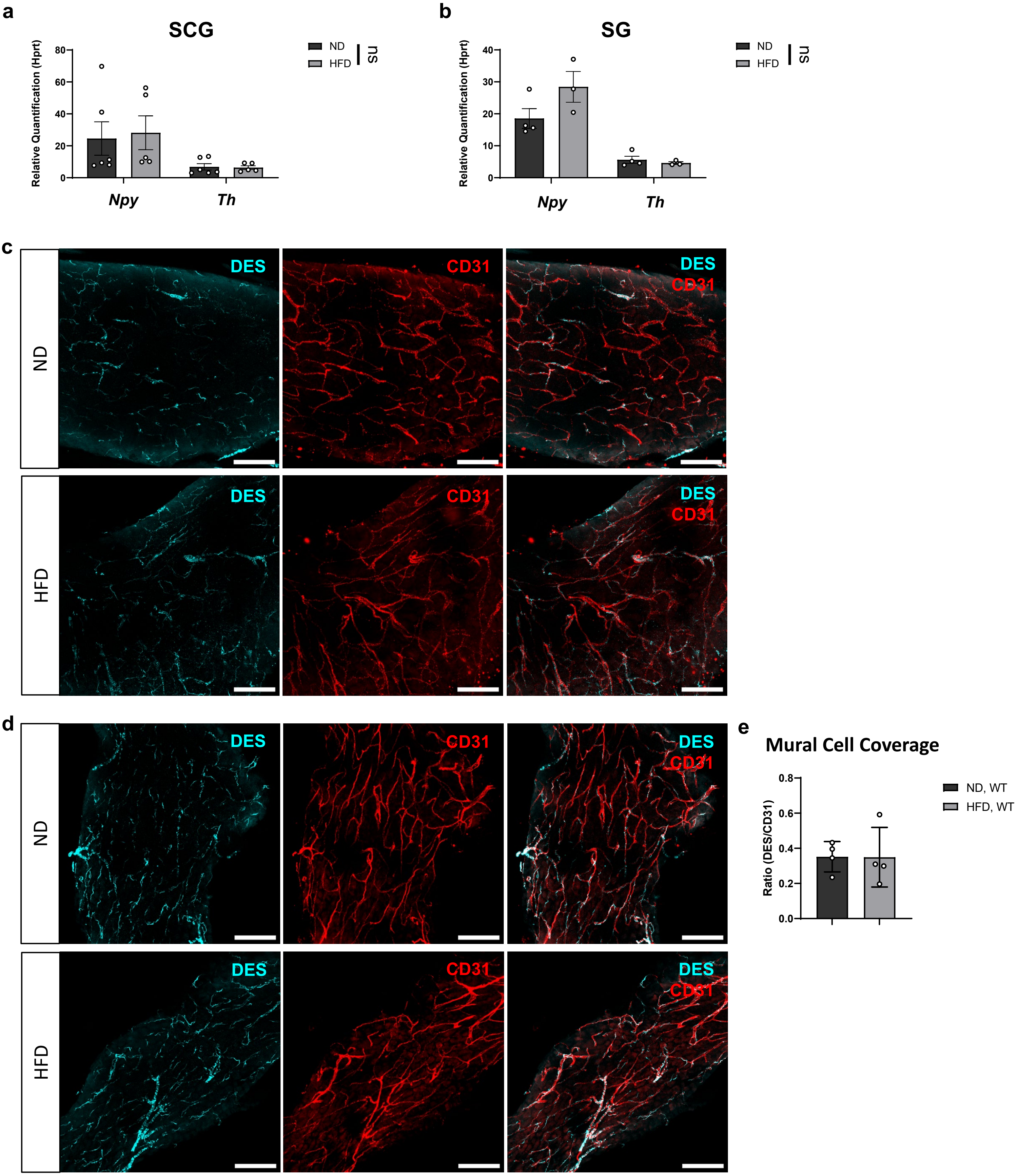

Supplementary Figure 5. Obesity does not affect *Npy* expression or mural cell coverage in sympathetic ganglia. **(a-b)** The expression of *Npy* and *Th* in the (a) superior cervical ganglia (SCG, n=6&5 mice) and (b) stellate ganglia (SG, n=4&3 mice) of ND- and HFD-treated 17-week-old WT male mice. **(c-d)** The images of (c) cleared SCG and (d) cleared SG of ND- and HFD-treated 17-week-old WT male mice stained with anti-DES (cyan) and anti-CD31 (red) antibodies. Scale bar=100μm. **(e)** The quantification of mural cell coverage in SG (n=4 mice). All values are expressed as mean ± SEM.

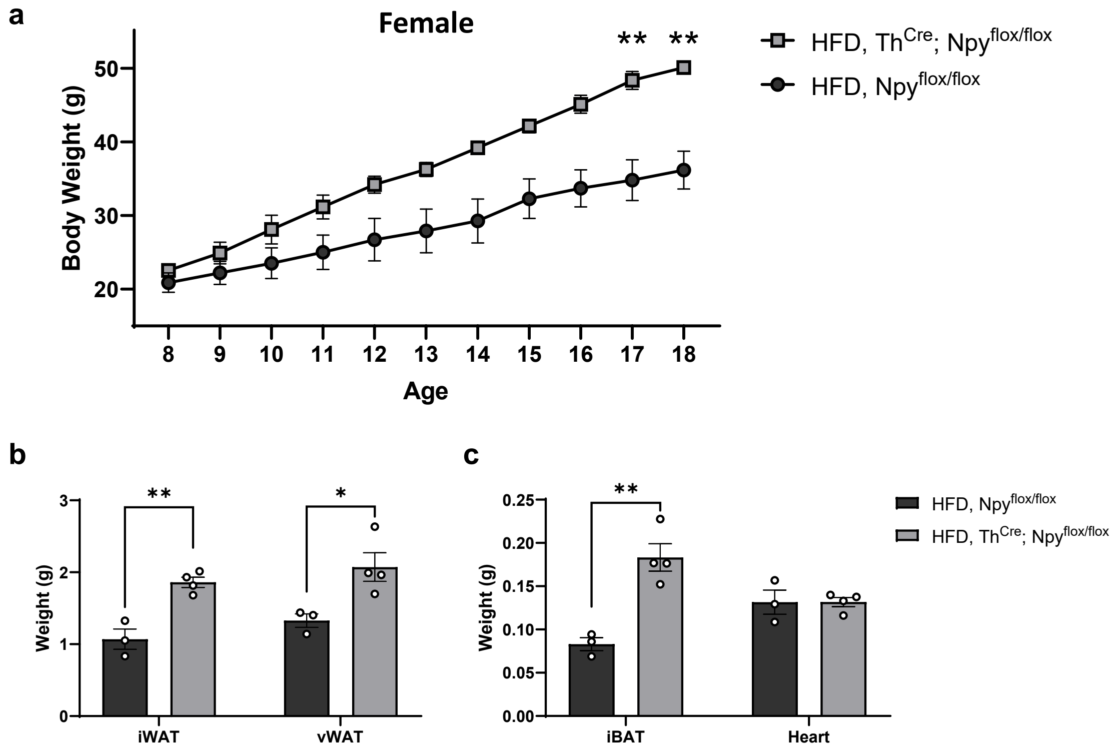

Supplementary Figure 6. Metabolic phenotype of female Th^{Cre}; $Npy^{flox/flox}$ mice is similar to that of males. **(a)** The weekly body weights of female Th^{Cre}; $Npy^{flox/flox}$ mice and WT mice treated with high-fat diet (HFD) (n=4). At Week 17, P=0.0041; at Week 18, P=0.0019. **(b-c)** The weights of (b) iWAT (P=0.0029) and vWATs (P=0.0299), and (c) iBAT (P=0.0039) and hearts of HFD-treated 18-week-old female Th^{Cre}; $Npy^{flox/flox}$ mice and $Npy^{flox/flox}$ mice (n=3&4). All values are expressed as mean ± SEM. Statistical comparisons were made using 2-tailed Student T-tests, *p<0.05, **p<0.01.

Supplementary Table 1. The Sequence of Primers for RT-qPCR

Gapdh	Forward	5'-AACTTTGGCATTGTGGAAGG-3'
	Reverse	5'-ACACATTGGGGGTAGGAACA-3'
Hprt	Forward	5'-CAGTCCCAGCGTCGTGATTA-3'
	Reverse	5'-TGGCCTCCCATCTCCTTCAT-3'
Plin1	Forward	5'-TAGAGTTCCTCCTGCCACCA-3'
	Reverse	5'-GTGCTGACCCTCCTCACAAG-3'
Pparg	Forward	5'-TCAAGGGTGCCAGTTTCG-3'
	Reverse	5'-GGAGGCCAGCATCGTGT-3'
Des	Forward	5'-TCCAGTCCTACACCTGCGAGAT-3'
	Reverse	5'-CGCAATGTTGTCCTGATAGCCA-3'
Npy	Forward	5'-CCCCAGAACAAGGCTTGAAG-3'
	Reverse	5'-TTGGAAAAGTCGGGAGAACAA-3'
Npy1r	Forward	5'-CTGATGGACCACTGGGTCTT-3'
	Reverse	5'-GAAGAAGCCACTGCAAGGAC-3'
Npy2r	Forward	5'-ATCATCTTGCTAGGGGTAGTTGG-3'
	Reverse	5'-CGGACCCATTTTCCACTCTCC-3'
Npy5r	Forward	5'-GTGAACTTTCTCATAGGCAACCT-3'
	Reverse	5'-GCATGATATGGCACATGGCTTT-3'
Pdgfrb	Forward	5'-AGGACAACCGTACCTTGGGTGACT-3'
	Reverse	5'-CAGTTCTGACACGTACCGGGTCTC-3'
Rgs5	Forward	5'-CAAAATGGCGGAGAAGGCAA-3'
	Reverse	5'-CCAAGTCAAAGCTGCGAGGA-3'
Tgfb1	Forward	5'-TGATACGCCTGAGTGGCTGTCT-3'
	Reverse	5'-CACAAGAGCAGTGAGCGCTGAA-3'
Cidea	Forward	5'-TGCTCTTCTGTATCGCCCAGT-3'
	Reverse	5'-GCCGTGTTAAGGAATCTGCTG-3'
Pgc1a	Forward	5'-CCCTGCCATTGTTAAGAC-3'
	Reverse	5'-TGCTGCTGTTCCTGTTTTC-3'
Ucp1	Forward	5'-ACTGCCACACCTCCAGTCATT-3'
	Reverse	5'-CTTTGCCTCACTCAGGATTGG-3'
Prdm16	Forward	5'- CAGCACGGTGAAGCCATT-3'
	Reverse	5'-GCGTGCATCCGCTTGTG-3'
Serca2b	Forward	5'-ACCTTTGCCGCTCATTTTCCAG-3'
	Reverse	5'-AGGCTGCACACACTCTTTACC-3'
Angpt1	Forward	5'-TGCAGCAACCAGCGCCGAAA-3'
	Reverse	5'-CACGGCAGTTCCCGTCGTGT-3'
Tnf	Forward	5'- ATGAGCACAGAAAGCATGA-3'
	Reverse	5'- TACAGGCTTGTCACTCGAA-3'
Il6	Forward	5'-ACGGCCTTCCCTACTTCACA-3'
	Reverse	5'- CATTTCCACGATTTCCCAGA-3'

References

1 Henriques, F. *et al*. Single-cell RNA profiling reveals adipocyte to macrophage signaling sufficient to enhance thermogenesis. *Cell reports* **32**, 107998 (2020).

2 Shamsi, F. *et al*. Vascular smooth muscle-derived Trpv1+ progenitors are a source of cold-induced thermogenic adipocytes. *Nature metabolism* **3**, 485-495 (2021).
