# Supplementary material for: Sympathetic neuropeptide Y protects from obesity by sustaining thermogenic fat
Source: Nature. 2024 Aug 28;634(8032):243–50. doi: 10.1038/s41586-024-07863-6 (PMC11446830; doi:10.1038/s41586-024-07863-6)
Supplement: Supplementary file 2 — Reporting Summary [file 41586_2024_7863_MOESM2_ESM.pdf]

nature portfolio

Corresponding author(s): Ana I. Domingos

Last updated by author(s): Jun 26, 2024

Reporting Summary

Nature Portfolio wishes to improve the reproducibility of the work that we publish. This form provides structure for consistency and transparency in reporting. For further information on Nature Portfolio policies, see our Editorial Policies and the Editorial Policy Checklist.

Statistics

For all statistical analyses, confirm that the following items are present in the figure legend, table legend, main text, or Methods section.

n/a	Confirmed	
☐	☒	The exact sample size (*n*) for each experimental group/condition, given as a discrete number and unit of measurement
☐	☒	A statement on whether measurements were taken from distinct samples or whether the same sample was measured repeatedly
☐	☒	The statistical test(s) used AND whether they are one- or two-sided<br>*Only common tests should be described solely by name; describe more complex techniques in the Methods section.*
☒	☐	A description of all covariates tested
☐	☒	A description of any assumptions or corrections, such as tests of normality and adjustment for multiple comparisons
☐	☒	A full description of the statistical parameters including central tendency (e.g. means) or other basic estimates (e.g. regression coefficient) AND variation (e.g. standard deviation) or associated estimates of uncertainty (e.g. confidence intervals)
☐	☒	For null hypothesis testing, the test statistic (e.g. *F*, *t*, *r*) with confidence intervals, effect sizes, degrees of freedom and *P* value noted<br>*Give P values as exact values whenever suitable.*
☒	☐	For Bayesian analysis, information on the choice of priors and Markov chain Monte Carlo settings
☒	☐	For hierarchical and complex designs, identification of the appropriate level for tests and full reporting of outcomes
☒	☐	Estimates of effect sizes (e.g. Cohen's *d*, Pearson's *r*), indicating how they were calculated

Our web collection on statistics for biologists contains articles on many of the points above.

Software and code

Policy information about availability of computer code

Data collection	EE and RER were measured using an indirect calorimetry system (Panlab; Harvard Apparatus; LE405 Gas Analyzer and Air Supply & Switching). Locomotor activity was measured using an LE001 PH Multitake Cage(Panlab; Harvard Apparatus) with a COMPULSE v1.0 Software (PanLab). Thermo-imaging data were recorded using Optris thermo-cameras (Optris PI 160 with standard 61 lens, Optris GmbH, Berlin, Germany) with an Optris PIX Connect software (rel. 2.0.6, Optris GmbH) or an FOTRIC 225s infrared camera (ED Fig.10l). Intravital 2 photon-microscopy was conducted with Optical microscope CARS (Coherent Anti-stokes Raman Scattering), TPEF (Two-photon excitation fluorescence) and Bright-field microscope, simultaneously, using confocal LSM 780-NLO Zeiss in the inverted microscope Axio Observer Z.1 (Carl Zeiss AG, Alemanha). Images were acquired using Zen Black v2.1 (Zeiss LSM880) and Inspector Pro (Miltenyi Biotec UltraMicroscope II Lightsheet Microscope). ELISA data were recorded using a FLUOstar Omega microplate reader. Flow cytometry data were collected using BD FACSDiva v6.0 (BD x20 Fortessa). qPCR data were acquired using StepOneTM qPCR system or a CFX96 Real-Time PCR Detection System (Bio-Rad) (ED Fig. 10m). Oxygen consumption of cultured adipocytes was measured using a Seahorse XF Analyzer.
Data analysis	EE and RER were analyzed using CalR Web-based Analysis Tool for Indirect Calorimetry Experiments (v1.3). Mouse body composition was recorded and analyzed using Minispec LF50 (Brucker). Thermo-image data in ED Fig. 10l were anlysed using FOTRIC software (v5.0.8.214). Images were analysed using Imaris v9.9.1, Fiji v1.53t and JaCoP v2.1.1. qPCR data were analysed using a StepOne Software v2.3. scRNA-seq datasets were analysed using Seurat v4.2.0 in an R v4.2.2 environment with the code deposited at Github (https://github.com/PLAVRVSO/scRNA-Seq-analysis-of-iBAT-SVF). Flow cytometry data were analysed using FlowJo v10.8.1. Statistical analysis was performed using GraphPad Prism v9.5.0.

For manuscripts utilizing custom algorithms or software that are central to the research but not yet described in published literature, software must be made available to editors and reviewers. We strongly encourage code deposition in a community repository (e.g. GitHub). See the Nature Portfolio guidelines for submitting code & software for further information.

Data

Policy information about availability of data

All manuscripts must include a data availability statement. This statement should provide the following information, where applicable:

- Accession codes, unique identifiers, or web links for publicly available datasets
- A description of any restrictions on data availability
- For clinical datasets or third party data, please ensure that the statement adheres to our policy

The public scRNA-Seq dataset of sympathetic neurons can be browsed on the Linnarson Lab website (https://linnarssonlab.org/sympathetic/), with raw data deposited on the Gene-Expression Omnibus (GEO) under the accession number GSE78845. The scRNA-Seq datasets of iWAT and iBAT were deposited on GEO under the accession numbers GSE154047 and GSE160585. The GWAS data of the association between genomic variants and metabolic traits is publicly available at Common Metabolic Diseases Knowledge Portal (https://hugeamp.org/). All the numeric data for supporting this research are available within the paper and the Supplementary information.

Human research participants

Policy information about studies involving human research participants and Sex and Gender in Research.

Reporting on sex and gender	N/A
Population characteristics	N/A
Recruitment	N/A
Ethics oversight	N/A

Note that full information on the approval of the study protocol must also be provided in the manuscript.

Field-specific reporting

Please select the one below that is the best fit for your research. If you are not sure, read the appropriate sections before making your selection.

☒ Life sciences ☐ Behavioural & social sciences ☐ Ecological, evolutionary & environmental sciences

For a reference copy of the document with all sections, see nature.com/documents/nr-reporting-summary-flat.pdf

Life sciences study design

All studies must disclose on these points even when the disclosure is negative.

Sample size	No statistical methods were used to pre-determine the sample sizes. The sample sizes were determined empirically based on previous studies and literature using the same experimental paradigm. (W Zeng et al. Cell 2015, Y Wang et al. Nature 2022)
Data exclusions	No data were excluded, except for mice with deteriorating health issues during the experiments or tissue samples contaminated.
Replication	All experiments are replicated at least twice with the same conclusion. All in vivo experiments were replicated in at least 2 independent cohorts. The exact numbers of replicated experiments are stated in each figure legend.
Randomization	Samples/animals were randomly allocated to experimental groups and proceeded in experiments.
Blinding	Data were collected and analysed blind, and post hoc registered to treatments and genotypes.

Reporting for specific materials, systems and methods

We require information from authors about some types of materials, experimental systems and methods used in many studies. Here, indicate whether each material, system or method listed is relevant to your study. If you are not sure if a list item applies to your research, read the appropriate section before selecting a response.

Materials & experimental systems

n/a	Involved in the study
☐	☒ Antibodies
☐	☒ Eukaryotic cell lines
☒	☐ Palaeontology and archaeology
☐	☒ Animals and other organisms
☒	☐ Clinical data
☒	☐ Dual use research of concern

Methods

n/a	Involved in the study
☒	☐ ChIP-seq
☐	☒ Flow cytometry
☒	☐ MRI-based neuroimaging

Antibodies

Antibodies used

The following primary antibodies were used for immunofluorescent staining: rat anti-CD31 (BioLegend, 102501 ,MEC13.3,1:100 dilution), rat anti-PLVAP (BioLegend, 120503, MECA32, 1:100 dilution), rabbit anti-DES (Abcam, AB15200, 1:500 dilution), rabbit anti-NPY (Cell Signalling, D7Y5A, 1:500 dilution), rabbit anti-NPY (Abcam, AB30914, 1:500 dilution), chicken anti-TH (Aves Labs, TYH73787982, 1:500 dilution), rabbit anti-TH (Sigma, Ab152, 1:500 dilution), mouse anti-NPY1R (Santa Cruz, sc-393192, 1:200 dilution), rat anti-PDGFRα (BioLegend, 135902, APA5, 1:200 dilution), rabbit anti-TAGLN (Abcam, AB14106, 1:250 dilution), Cy3 anti-αSMA (Sigma, C6198, 1A4, 1:250 dilution), goat anti-SOX17 (R&D, AF1924, 1:250), goat anti-EPHB4 (R&D, AF3034, 1:250), rabbit anti-UCP1 (Abcam, Ab10983, 1:500).
The following antibodies were used for FACS and flow cytometry: AF700 anti-CD45 (BioLegend, 103128), BUV395 anti-CD45 (BD Horizon, 564279), Pacific Blue anti-CD31 (BioLegend, 102421), APC anti-PDGFRa (BioLegend 135907), AF488 anti-NG2 (Sigma, MAB5384A4), and AF488 anti-DES (Abcam, AB185033, Y66). All antibodies for FACS and flow cytometry were diluted at 1:500. LIVE/DEAD™ Fixable Near-IR Dead Cell Stain Kit (1:1000, ThermoFisher, L10119) was used for live/dead staining.
APC anti-CD104b antibody (1:100, BioLegend, 136007) was used for magnetic sorting.

Validation

Rat anti-CD31 (BioLegend, 102501 ,MEC13.3): validated in mouse tissues by K Cheung et al. PNAS 2015, E5815–E5824. https://doi.org/10.1073/pnas.1509627112
Rat anti-PLVAP (BioLegend, 120503, MECA32): validated in mouse tissues by S Carloni et al. Science 2021, 374(6566), 439–448. https://doi.org/10.1126/science.abc6108
Rabbit anti-DES (Abcam, AB15200): validated in mouse tissues by W Yang et al. Hepatology 2021 and J Chang et al. Nature Medicine 2017, 23(4), 450–460. https://doi.org/10.1038/nm.4309
Rabbit anti-NPY (Cell Signalling, D7Y5A): validated in mouse tissues by Rahman T. U. et al. Oncotarget, 8(32), 53450–53464. https://doi.org/10.18632/oncotarget.18519
Chicken anti-TH (Aves Labs, TYH73787982): validated in mouse tissues by B. Sofia Beas et al. Nature communications, 11(1), 6218. https://doi.org/10.1038/s41467-020-19980-7
Rabbit anti-TH (Sigma, Ab152): validated in mouse tissues by D. Rycko et al. PNAS 2013, 110(34), E3235–E3242. https://doi.org/10.1073/pnas.1301125110
Mouse anti-NPY1R (Santa Cruz, sc-393192): validated in mouse tissues by K Xu et al. Wound Repair and Regeneration 2018 and X. Kang et al. American Society for Bone and Mineral Research, 35(7), 1375–1384. https://doi.org/10.1002/jbmr.3991
Rat anti-PDGFRα (BioLegend, 135902, APA5): validated in mouse tissues by MW Hogarth et al. Nature Communication 2019, 10(1), 2430. https://doi.org/10.1038/s41467-019-10438-z
Rabbit anti-TAGLN (Abcam, AB14106): validated in mouse tissues by C. De Bono et al. Nature communications, 14(1), 1551. https://doi.org/10.1038/s41467-023-37015-9
Cy3-conjugated anti-αSMA (Sigma, C6198): validated in mouse tissues by S. Ock, Cell death & disease, 12(7), 688. https://doi.org/10.1038/s41419-021-03965-5
Goat anti-SOX17 (R&D, AF1924) and Goat anti-EPHB4 (R&D, AF3034, 1:250) are validated in mouse tissues by M. Corada et al. Nature communications, 4, 2609. https://doi.org/10.1038/ncomms3609
Rabbit anti-UCP1 (Abcam, Ab10983): validated in mouse tissues by Y. Oguri et al. Cell, 182(3), 563–577.e20. https://doi.org/10.1016/j.cell.2020.06.021
AF700 anti-CD45 (BioLegend, 103128), Pacific Blue anti-CD31 (BioLegend, 102421), and APC anti-PDGFRa (BioLegend 135907) are validated in our previous paper by E. Habermann et al. Immunity, 57(1), 141–152.e5. https://doi.org/10.1016/j.immuni.2023.11.006
Other conjugated antibodies have been validated by the commercial vendors with information demonstrated on their websites:
BUV395 anti-CD45 (BD Horizon, 564279): https://www.bdbiosciences.com/en-gb/products/reagents/flow-cytometry-reagents/research-reagents/single-color-antibodies-ruo/buv395-rat-anti-mouse-cd45.564279
AF488 anti-NG2 (Sigma, MAB5384A4): https://www.sigmaaldrich.com/GB/en/product/mm/mab5384a4
AF488 anti-DES (Abcam, AB185033, Y66): https://www.abcam.com/en-gb/products/primary-antibodies/alexa-fluor-488-desmin-antibody-y66-cytoskeleton-marker-ab185033
APC anti-CD104b antibody (1:100, BioLegend): https://www.biolegend.com/en-gb/products/apc-anti-mouse-cd140b-antibody-6441

Eukaryotic cell lines

Policy information about cell lines and Sex and Gender in Research

Cell line source(s)

3T3-L1 cell line was established and described in a previously published report (H Green et al. Cell 1975) and was a gift from Robin Klemm (CAC Freyre et al. Molecular Cell 2019, from American Type Culture Collection). RAW264.7 was purchased from Sigma (Sigma 91062702-1VL).

Authentication

No further authentication was performed for 3T3-L1 and RAW264.7.

Mycoplasma contamination

No further test for Mycoplasma was performed for 3T3-L1 and RAW264.7.

Commonly misidentified lines (See ICLAC register)	No commonly misidentified lines were used.

Animals and other research organisms

Policy information about studies involving animals; ARRIVE guidelines recommended for reporting animal research, and Sex and Gender in Research

Laboratory animals	ThCre mice (B6.Cg-7630403G23RikTg(Th-cre)1Tmd/J; stock no. 008601), Cx3cr1GFP/+ mice (Cx3cr1tm1Litt/LittJ; stock no. 008451) were purchased from the Jackson Lab (JAX). Npyflox/flox mice were a donation from Ivo Kalajzic Lab at the Department of Reconstructive Science, University of Connecticut45 under MTA. Tissues of NPY-GFP mice (B6.FVB-Tg(Npy-hrGFP)1Lowl/J) are from Tamas Horvath Lab at Brandy Memorial Laboratory, Yale University. Tissue of Npy1rCre; Rosa26tdTomato mice were from Professor Michael Roberts at Department of Otolaryngology-Head and Neck Surgery, University of Michigan. Sympathetic neuron-specific NPY-cKO mice were generated by crossing ThCre mice with Npyflox/flox mice. Diet-induced obesity (DIO) was achieved by feeding mice an HFD (Diet Research, D12492) when they were 7 weeks old, and lasted for 10 weeks. The body weight of each mouse and the food consumption in each cage were recorded weekly. All the mice were group housed in standard housing at controlled room temperature (21-23ºC) and 50% humidity under a 12-12-hour light-dark cycle and given access to diet and water ad libitum, and.
Wild animals	No wild animals were used in this study.
Reporting on sex	Both sexes were used for in vivo studies. The sexes of mice were indicated in each figure legend.
Field-collected samples	No field-collected samples were used in this study.
Ethics oversight	All experimental procedures were performed on living animals in accordance with the United Kingdom ANIMALS ACTS 1986 under the project license (PPL number: P80EDA9F7) and personal licenses granted by the United Kingdom Home Office. Ethical Approval was provided by the Ethical Review Panel at the University of Oxford.

Note that full information on the approval of the study protocol must also be provided in the manuscript.

Flow Cytometry

Plots

Confirm that:

☒ The axis labels state the marker and fluorochrome used (e.g. CD4-FITC).

☒ The axis scales are clearly visible. Include numbers along axes only for bottom left plot of group (a 'group' is an analysis of identical markers).

☒ All plots are contour plots with outliers or pseudocolor plots.

☒ A numerical value for number of cells or percentage (with statistics) is provided.

Methodology

Sample preparation	Dissected iWATs and BATs were minced and digested in the enzyme mixture (for each sample, 500µL Collagenase II (4 mg/mL, C6885), 500µL Hyaluronidase (5.3 mg/mL=40000 U/mL, H3884), and 5 µL Dnase I (BioLabs, M0303L)) in a 37 ℃-water bath shaking for 45 minutes, and samples were pipetted every 10 minutes. Digestion was stopped by adding FACS buffer (PBS containing 2% FBS), and single-cell suspension was collected by filtering the digested sample using EASYStrainer cell sieves with 70µm mesh (Greiner, 542070). To prepare the samples for sorting or flow cytometry, cells were first treated with red-blood-cell lysis buffer (BioLegend, 420301) to remove red-blood cells and Fc block (ThermoFisher, 14-9161-73) before staining with antibodies. Before immunolabelling for intracellular markers, cells were fixed and permeabilised using the eBioscience Intracellular Fixation and Permeabilization Buffer Set (ThermoFisher, 88-8824-00).
Instrument	BD FACSAria III sorter or LSRFortessa X20 cytometer.
Software	Flow cytometry data were collected using BD FACSDiva v6.0 and analysed using FlowJo v10.8.1.
Cell population abundance	At least 10000 singlet cells were harvested.
Gating strategy	For all experiments, the SSC-A/FSC-A gating was used to find viable cells in the starting cell population, and singlet cells were identified using the FSC-H/FSC-W gating. Isotype controls were used to distinguish marker-positive events from background events.

☒ Tick this box to confirm that a figure exemplifying the gating strategy is provided in the Supplementary Information.
